# Supplementary material for: Factors Associated with Colostrum Quality, the Failure of Transfer of Passive Immunity, and the Impact on Calf Health in the First Three Weeks of Life
Source: Animals (Basel). 2023 May 24;13(11):1740. doi: 10.3390/ani13111740 (PMC10251921; doi:10.3390/ani13111740)
Supplement: Supplementary file 1 [file animals-13-01740-s001.zip › animals-2414628-supplementary.pdf]

Table S1: Part 1: Overview on the physical examination of 250 calves between the 3rd and the 6th days of age. The physical examination was carried out according to Baumgartner and Wittek 2018.

Clinical assessment	N calves	% calves
**General behavior**		
Normal	203	81.2
Slightly depressed	43	17.2
Moderately depressed	3	1.2
Severely depressed	1	0.4
**Posture**		
Normal	227	90.8
Arched back	15	6.0
Extended/sawhorse like	1	0.4
Other	7	2.8
**Body condition**		
Very good	236	94.4
Good	14	5.6
**Hair and skin assessment**		
Smooth, shiny, flat lying	229	91.6
Shaggy	19	7.6
Alopecia	1	0.4
Missing	1	0.4
**Skin turgor**		
Normal	234	93.6
Skin fold remains	1	0.4
Slight enophthalmus	4	1.6
Moderate enophtalmus	1	0.4
Missing	10	4.0
**Navel palpation**		
Normal	225	90.0
Enlarged	8	3.2
Enlarged and painful	8	3.2
Enlarged, painful and rough	9	3.6
**Mucus membranes**		
Normal	244	97.6
Anaemia	5	2.0
Cyanosis	1	0.4
Icterus	0	0
Reddened	0	0
**Nasal discharge**		
No discharge	221	88.4
Serous	27	10.8
Mucous	2	0.8
Pus	0	0

Table S2: Part 2: Overview on the physical examination of 250 calves between the 3rd and the 6th days of age. The physical examination was carried out according to Baumgartner and Wittek 2018.

Clinical assessment	N calves	% calves
**Mucus membranes**		
Normal	244	97.6
Anaemia	5	2.0
Cyanosis	1	0.4
**Nasal discharge**		
No discharge	221	88.4
Serous	27	10.8
Mucous	2	0.8
**Coughing**		
No coughing	226	90.4
Intermitted coughing	12	4.8
Missing	12	4.8
**Lung auscultation**		
Normal	190	76.0
Moderate aggravated breathing sounds	57	22.8
Focal no respiration sounds	1	0.4
Missing	2	0.8
**Heart sounds**		
Normal	244	97.6
Heart murmur	6	2.4
**Abdominal auscultation**		
Intestine contraction normal	224	89.6
Reduced contraction	4	1.6
Increased contraction	22	8.8
**Swing and percussion auscultation**		
Negative right and left	250	100
**Amount of faeces**		
+	149	59.6
++	92	36.8
+++	9	3.6
**Faecal color**		
Grey	1	0.4
Yellow	58	23.2
Ochre	130	52.0
Brown	59	23.6
Reddish	1	0.4
Greenish	1	0.4
**Faecal consistency**		
Normal	233	93.2
Fluid	9	3.6
Watery	5	2.0
Dry	3	1.2
**Faecal admixture**		
No	245	98.0
Yes	5	2.0

Table S3: Binary logistic regression analysis was carried out using the serum Brix levels as dependant variable. The table gives an overview on the association of diarrhea, bovine respiratory disease (BRD), navel illness, diarrhea and BRD and an abnormal general behavior with a low serum Brix level in the first week of life. Odds ratios (OR) were calculated using the basis category = healthy calves (OR = 1) as reference.

Factor	N calves total	Brix <8.4 %		Brix ≥8.4 %		OR (95 % CI)
		N calves	% calves	N calves	% calves	
**Diarrhea**						
Healthy	215	74	34.4	141	65.6	1
Moderate diarrhea	24	13	54.2	11	45.8	2.25 (0.96-5.26)
Severe diarrhea	11	6	54.5	5	45.5	2.29 (0.68-7.75)
**BRD**						
Healthy	234	87	37.2	147	62.8	1
Moderate pneumonia	9	3	33.3	6	66.7	0.84 (0.21-3.46)
Severe pneumonia	7	3	42.9	4	57.1	1.27 (0.28-5.78)
**Navel illness**						
Healthy	249	92	36.9	157	63.1	n. a.
Moderate enlarged navel	0					
Severely enlarged navel	1	1	100	0	0	
**Diarrhea and BRD**						
Healthy	246	92	34.7	154	62.6	n. a.
Moderate	2	0	0	2	100	
Severe	2	1	50.0	1	50.0	
**Abnormal general behavior**						
Healthy	230	82	35.7	148	64.3	1
Moderate	16	10	62.5	6	37.5	3.05 (1.05-8.55)
Severe	4	1	25.0	3	75.0	0.60 (0.06-5.88)

Table S4: Binary logistic regression analysis was carried out using the serum Brix levels as dependant variable. The table gives an overview on the association of diarrhea, bovine respiratory disease (BRD), navel illness, diarrhea and BRD and an abnormal abnormal general behavior with a low serum Brix level in the first week of life. Odds ratios (OR) were calculated using the basis category = healthy calves (OR = 1) as reference. Moderate and severe disease events were summarized as having the disease (yes) or not having the disease (healthy). All statistically significant values ($p < 0.05$) are highlighted with an asterix *.

Factor	N calves total	Brix <8.4 %		Brix ≥8.4 %		OR (95 % CI)
		N calves	% calves	N calves	% calves	
**Diarrhea**						
Healthy	215	74	34.4	141	65.6	1
Diarrhea	35	19	54.3	16	45.7	2.26 (1.1-4.65)*
**BRD**						
Healthy	234	87	37.2	147	62.8	1
Pneumonia	16	6	37.5	10	62.5	1.01 (0.36-2.89)
**Navel illness**						
Healthy	249	92	36.9	157	63.1	n. a.
Navel illness	1	1	100	0	0	
**Diarrhea and BRD**						
Healthy	246	92	34.7	154	62.6	1
Diarrhea + Resp. Disease	4	1	25.0	3	75.0	0.56 (0.06-5.44)
**Abnormal general behavior**						
Healthy	230	82	35.7	148	64.3	1
Abnormal	20	11	55.0	9	45.0	2.21 (0.88-5.56)

Table S5: Binary logistic regression analysis was carried out using the serum Brix levels as dependant variable. The table gives an overview on the association of diarrhea, bovine respiratory disease (BRD), navel illness, diarrhea and BRD and an abnormal general behavior with a low serum Brix level in the second week of life. Odds ratios (OR) were calculated using the basis category = healthy calves (OR = 1) as reference. All statistically significant values (p < 0.05) are highlighted with an asterix *.

Factor	N calves total	Brix <8.4 %		Brix ≥8.4 %		OR (95 % CI)
		N calves	% calves	N calves	% calves	
**Diarrhea**						
Healthy	209	69	33.0	140	67.0	1
Moderate diarrhea	24	12	50.0	12	50.0	2.03 (0.87-4.74)
Severe diarrhea	12	9	75.0	3	25.0	6.1 (1.6-23.26)*
**BRD**						
Healthy	234	84	37.2	147	62.8	
Moderate pneumonia	4	0	0	4	100.0	n. a.
Severe pneumonia	7	3	42.9	4	57.1	
**Navel illness**						
Healthy	244	90	36.9	154	63.1	
Moderate enlarged navel	0					n. a.
Severely enlarged navel	1	0	0	1	100.0	
**Diarrhea and BRD**						
Healthy	240	89	37.1	151	62.9	
Moderate	3	0	0	3	100	n. a.
Severe	2	1	50.0	1	50.0	
**Abnormal general behavior**						
Healthy	230	83	36.1	147	63.9	
Moderate	14	7	50.0	7	50.0	n. a.
Severe	1	0	0	1	100.0	

Table S6: Binary logistic regression analysis was carried out using the serum Brix levels as dependant variable. The table gives an overview on the association of diarrhea, bovine respiratory disease (BRD), navel illness, diarrhea and BRD and an abnormal general behavior with a low serum Brix level in the second week of life. Odds ratios (OR) were calculated using the basis category = healthy calves (OR = 1) as reference. Moderate and severe disease events were summarized as having the disease (yes) or not having the disease (healthy). All statistically significant values ($p < 0.05$) are highlighted with an asterix*.

Factor	N calves total	Brix <8.4 %		Brix ≥8.4 %		OR (95 % CI)
		N calves	% calves	N calves	% calves	
**Diarrhea**						
Healthy	209	69	33.0	140	67.0	1
Diarrhea	36	21	58.3	15	41.7	2.84 (1.38-5.85)*
**BRD**						
Healthy	234	84	37.2	147	62.8	1
Pneumonia	11	3	27.3	8	72.7	0.63 (0.16-2.45)
**Navel illness**						
Healthy	244	90	36.9	154	63.1	n. a.
Navel illness	1	0	0	1	100.0	
**Diarrhea and BRD**						
Healthy	240	89	37.1	151	62.9	1
Diarrhea + Resp. Disease	5	1	20.0	4	80.0	0.42 (0.04-3.86)
**Abnormal general behavior**						
Healthy	230	83	36.1	147	63.9	1
Abnormal	15	7	46.7	8	53.3	1.55 (0.54-4.42)

Table S7: Binary logistic regression analysis was carried out using the serum Brix levels as dependant variable. The table gives an overview on the association of diarrhea, bovine respiratory disease (BRD), navel illness, diarrhea and BRD and an abnormal abnormal general behavior with a low serum Brix level in the third week of life. Odds ratios (OR) were calculated using the basis category = healthy calves (OR = 1) as reference. All statistically significant values ($p < 0.05$) are highlighted with an asterix*.

Factor	N calves total	Brix <8.4 %		Brix ≥8.4 %		OR (95 % CI)
		N calves	% calves	N calves	% calves	
**Diarrhea**						
Healthy	211	70	33.2	141	66.8	1
Moderate diarrhea	14	8	57.1	6	42.9	2.69 (0.90-8.06)
Severe diarrhea	9	8	88.9	1	11.1	16.13 (1.98-125.0)*
**BRD**						
Healthy	231	85	36.8	146	63.2	n. a.
Moderate pneumonia	2	0	0	2	100.0	
Severe pneumonia	1	1	100.0	0	0	
**Navel illness**						
Healthy	233	86	36.9	147	63.1	n. a.
Moderate enlarged navel	1	0	0	1	100.0	
Severely enlarged navel	0	0	0	0	0	
**Diarrhea and BRD**						
Healthy	231	85	36.8	146	63.2	n. a.
Moderate	2	0	0	2	100.0	
Severe	1	1	100.0	0	0	
**Abnormal general behavior**						
Healthy	228	82	36.0	146	64.0	n. a.
Moderate	6	4	66.7	2	33.3	
Severe	0	0	0	0	0	

Table S8: Binary logistic regression analysis was carried out using the serum Brix levels as dependant variable. The table gives an overview on the association of diarrhea, bovine respiratory disease (BRD), navel illness, diarrhea and BRD and an abnormal general behavior with a low serum Brix level in the third week of life. Odds ratios (OR) were calculated using the basis category = healthy calves (OR = 1) as reference. Moderate and severe disease events were summarized as having the disease (yes) or not having the disease (healthy).

**Factor**	**N calves total**	**Brix <8.4 %**		**Brix ≥8.4 %**		**OR (95 % CI)**
		**N calves**	**% calves**	**N calves**	**% calves**	
**Diarrhea**						
Healthy	211	70	33.2	141	66.8	1
Diarrhea	23	16	69.6	7	30.4	4.61 (1.81-11.76)*
**BRD**						
Healthy	231	85	36.8	146	63.2	1
Pneumonia	3	1	33.3	2	66.7	0.86 (0.08-9.62)
**Navel illness**						
Healthy	233	86	36.9	147	63.1	n. a.
Navel illness	1	0	0	1	100.0	
**Diarrhea and BRD**						
Healthy	231	85	36.8	146	63.2	1
Diarrhea + Resp. Disease	3	1	33.3	2	66.7	0.86 (0.08-9.62)
**Abnormal general behavior**						
Healthy	228	82	36.0	146	64.0	1
Abnormal	6	4	66.7	2	33.3	3.56 (0.64-20.0)

Kälberkarte für das Projekt „Kolostrumversorgung von Kälbern im Bundesland Salzburg und Validierung eines Immunglobulin-Schnelltests“

LFBIS-Nr. Tierhalter:							

Ohrmarke Kalb: AT __ __ __ __ __ __ __ __ __

Tab. 1: Bitte vom 1.-7. Lebenstag ausfüllen

Lebenstag	1			2			3			4			5			6			7		
Art der Tränke[1]																					
Uhrzeit der Tränke																					
Trinkmenge																					
Allgemeinbefinden[2]																					
Kotkonsistenz[3]																					
Atmung[4]																					
Husten[5]																					
Nasenausfluss[6]																					
Nabel[7]																					
Sonstige Erkrankung																					
Besondere Vorkommnisse[8]																					

Tab. 2: Bitte vom 8.-28. Lebenstag ausfüllen

Lebenstag	8	9	10	11	12	13	14	15	16	17	18	19	20	21	22	23	24	25	26	27	28
Art der Tränke[1]																					
Trinkmenge																					
Allgemeinbefinden[2]																					
Kotkonsistenz[3]																					
Atmung[4]																					
Husten[5]																					
Nasenausfluss[6]																					
Nabel[7]																					
Sonstige Erkrankung																					
Besondere Vorkommnisse[8]																					

Legende auf der Rückseite!

Legende:

1: MAT= Milchaustauscher; V= Vollmilch; S= angesäuerte Milch; K=Kolostrum; T= Transitmilch; J= Joghurttränke; (Sonstige Tränken bitte selbstständig ausfüllen)

2: leeres Feld= ungestört; -=Kalb wirkt müde (ggr. verm.); --=festliegend (mgr. verm.); ---=komatös (hgr. verm.)

3: leeres Feld=normal; B= dünnbreiig; W= wässrig

4: leeres Feld =ungestört; +=Kalb atmet schneller; ++=Kalb atmet wesentlich schneller („hechelt"); +++=Kalb hat Atemnot

5: leeres Feld= ungestört; S=stoßweise (<3 mal); A=anfallsweise (>3 mal);

6: leeres Feld= ungestört; S=serös; M=mukös; E=eitrig

7: leeres Feld= ungestört (ca. 1cm dick); += zweifinger dick; ++= dreifinger dick; +++=handdick

8: Bitte besondere Vorkommnisse, wie zum Beispiel Enthornung, Kastration, Impfung, Umstallung etc. dokumentieren
